# Genetic surveillance of *Plasmodium–Anopheles* compatibility markers during *Anopheles stephensi* associated malaria outbreak

**DOI:** 10.1186/s13071-025-06981-y

**Published:** 2025-08-20

**Authors:** Elizabeth Waymire, Dejene Getachew, Isuru Gunarathna, Joseph Spear, Grace Lloyd, Madison Follis, Avery A. Kaye, Said Ali, Solomon Yared, Tamar E. Carter

**Affiliations:** 1 Department of Biology, Baylor University, Waco, TX USA; 2 Department of Applied Biology, Adama Science and Technology University, Adama, Ethiopia; 3 National Malaria Control Program, Ministry of Health Development, Hargeisa, Somaliland; 4 Department of Biology, Jigjiga University, Jijiga, Ethiopia

**Keywords:** Malaria outbreak, *Pfs47*, *Pvs47*, *Pv47*, *P47Rec*, Vector-parasite interaction, *Anopheles stephensi*

## Abstract

**Background:**

Despite a previous decline in malaria in Ethiopia, an outbreak in Dire Dawa in 2022 implicated the invasive vector *Anopheles stephensi* as responsible. The efficient transmission of *Plasmodium* by invasive *An. stephensi* raises questions about the molecular basis of compatibility between parasite and vector, and the origin of the *Plasmodium* being transmitted. The *Plasmodium P47* gene is involved in parasite–vector interactions in the mosquito, and along with the corresponding mosquito *P47* receptor (*P47Rec*), can be critical in the establishment of *Plasmodium* infections in anophelines.

**Methods:**

Herein, we analyzed *P47* and *P47Rec* sequences to determine the origin of *Plasmodium* detected in *An. stephensi* during the outbreak and evaluate markers of compatibility. This was completed using polymerase chain reactions and Sanger sequencing.

**Results:**

Of 160 mosquitoes screened, 6.21% of the mosquitoes screened were positive for *P. falciparum* DNA and 4.37% were positive for *P. vivax* DNA. Analysis of geographically informative SNPs at positions 707 and 725 in *Pfs47* revealed that these *P. falciparum* strains only exhibit the African haplotype. Minimum spanning network (MSN) analysis revealed connectivity between *Pfs47* in Dire Dawa and *Pfs47* sequences in Africa, further supporting that these *Plasmodium* strains are of African origin. We also evaluated the connectivity between *Pv47* in this study and African and Asian *Pv47* using MSN analysis. *Pv47* in both continents displayed shared haplotypes, suggesting little differentiation between the African and Asian strains in *P. vivax*. Lastly, we identified a single amino acid change in the P47Rec within *An. stephensi*, which could act as a marker for the propensity of *An. stephensi* populations to outbreak.

**Conclusions:**

Overall, these results provide evidence of African *P. falciparum* in invasive *An. stephensi* and identify *P47Rec* as a potential marker, which could be applied as a molecular diagnostic for propensity for an outbreak. The relatively high frequencies of *Plasmodium* parasites observed in *An. stephensi* may suggest that this mosquito species contributed to the malaria outbreak. Our findings lay the groundwork for further research into the interactions between the invasive mosquito species *An. stephensi* and African *Plasmodium* strains, with the goal of predicting future outbreaks.

**Graphical Abstract:**

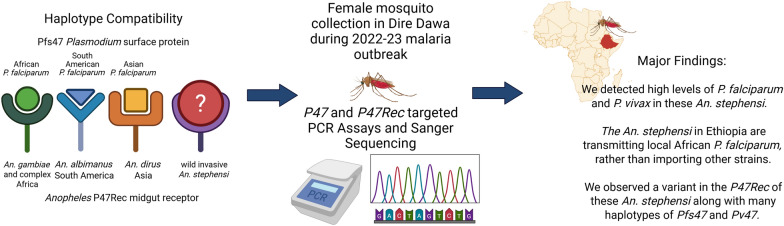

**Supplementary Information:**

The online version contains supplementary material available at 10.1186/s13071-025-06981-y.

## Background

*Anopheles stephensi* was first identified in Kebridehar in southeastern Ethiopia in 2016 and was detected across eastern Ethiopia soon after in 2018 [1, 2]. Rapid growth occurred across the country between 2010 and now, with multiple cities such as Jijiga and Dire Dawa experiencing urbanization [3–5]. This rapid urbanization created ideal conditions for *An. stephensi* oviposition, allowing the invasive populations to grow [6]. This is exemplified by a malaria outbreak in 2022 in Dire Dawa, where approximately 2400 cases were reported. This outbreak continued well into the dry season, demonstrating the ability of this invasive vector to thrive in urban environments and in unfavorable climates, transmitting both *P. falciparum* and *P. vivax* [7, 8].

The *Plasmodium* gametocyte surface antigen 47 (*P47)* is a gene present in female *P. falciparum* and *P. vivax*. It encodes for the P47 ligand on the surface of *Plasmodium* parasites expressed during the gametic and ookinete stage in the mosquito mid-gut [9–11]. This protein is critical for parasite–vector interactions, allowing the parasite to survive the mosquito immune response and continue its infection cycle. Previous studies have revealed that the genetic diversity of the *P. falciparum P47* gene (*Pfs47*) is structured geographically on a continental level due to the natural selection imposed on *P. falciparum* by evolutionarily distant vectors, specifically between *Pfs47* and the mosquito receptor (P47Rec) [12]. This molecular interaction has been described as a “lock-and-key" relationship [13]. Two single nucleotide polymorphisms (SNPs) resulting in nonsynonymous amino acid substitutions have been identified at base pair positions 707 and 725 of the gene. The predominant haplotypes at these loci differ between Africa, Asia, and South America, with 98–99% of individuals within each continent sharing the same haplotype [12]. These SNPs occur in domain 2 of Pfs47, which is the most polymorphic region of the protein, and amino acid changes in this region define compatibility between selected parasite strains and vector species, with specificity between each continent’s parasites and vectors [12, 14].

*Plasmodium vivax* evasion of the *Anopheles* immune system and parasite–vector compatibility mediation by the *P. vivax P47* gene (*Pv47*) is less understood. There are limited amino acid substitutions of the cysteine residues in Pv47, indicating a possible target for transmission blocking vaccines (TBVs). Genome-wide population analysis of *Pv47* demonstrated a clear differentiation between the eastern hemisphere and the western hemisphere parasite populations, where South American *P. vivax* populations are genetically distinct from Asia. Only two African countries were represented in this study, Madagascar and Mauritania, which clustered with Indian *Pv47* [15]. A previous study cemented this idea of South American *Pv47* being very distinct from Asian and African *Pv47*; however, Africa shares *Pv47* haplotypes with Asia, and minimum spanning network (MSN) analysis demonstrates that the two are genetically closer than the South American haplotypes [16].

The *P47–P47Rec* interactions in *An. stephensi* are not fully understood, but previous findings hint that the specificity of compatibility may differ than what is observed in *An. gambiae*. A compatibility study of amino acid variation at *Pfs47* in African and South American strains revealed that single amino acid substitutions in *Pfs47* determined *P. falciparum* infection in *An. gambiae* [17], while in *An. stephensi* SDA500, *P. falciparum* infection occurred regardless of the amino acid substitutions, even without *Pfs47* entirely. These findings suggest that the *Pfs47*–*P47Rec* interaction is less specific or is less deterministic in this *An. stephensi* strain compared with other *Anopheles* species [17]. The strain of *An. stephensi* used in the study was the selectively bred SDA500 strain, bred to be highly susceptible to *P. falciparum* [18]. Because of this evidence, it is possible that some vectors may be naturally more permissive to many *Pfs47* haplotypes [9]. In addition, a previous study demonstrated that the P47 receptor is 100% conserved in anophelines such as the *An. gambiae* complex and *An. funestus* [19]. However, there were two clades in the phylogenetic analysis representing *An. stephensi*, and Ethiopian P47Rec fell within the same clade as the receptor from SDA500 [19]. Ultimately, this system is not well studied in wild *An. stephensi* populations, where there is a critical knowledge gap in the interactions of this invasive vector and its parasite.

Through leveraging the P47/P47Rec system we can: (1) provide vital information regarding the diversity of these strains in eastern Ethiopia for the possibility of using P47-based TBVs in these populations, (2) reveal the importation of clinically relevant *P. falciparum* from South Asia in this invasive vector, and (3) uncover key genetic signatures that could function as a predictor for propensity to outbreak. This study, therefore, investigated the sequence diversity of *Pfs47* and *Pv47* in wild *An. stephensi,* during the outbreak in Dire Dawa, nucleotide and amino acid sequences of *Pv47* in *An. stephensi* to determine SNPs or amino acids that separate continental origin, and P47Rec sequences in wild *An. stephensi* to probe intraspecies diversity and gain further insight into the *Plasmodium–An. stephensi* relationship.

## Methods

### Description of study site and outbreak

The outbreak of malaria began in Dire Dawa, Ethiopia, in late November 2021, continuing into the dry season of 2022, ultimately concluding around May [8, 20]. Dire Dawa is located 515 km east of Addis Ababa in central eastern Ethiopia [21]. Dire Dawa University is located in the northwest corner of Dire Dawa, with both institutional buildings, a health clinic, and dormitories on campus [8]. Mosquitoes were collected specifically at Dire Dawa University, where there seemed to be a concentration of cases [8].

### Collection of *Anopheles stephensi*

*Anopheles stephensi* mosquitoes were collected in Dire Dawa, Ethiopia, between 19 and 31 March 2022, using a mouth aspirator. Adult mosquitoes were caught from inside offices or outdoors in manholes. Mosquitoes were then sorted and categorized as culicines and anophelines. The latter were identified according to a morphological key [22]. *An. stephensi* mosquitoes were sexed, and females stored individually in 1.5 mL tubes in a bag containing silica gel. Blood fed mosquitoes were recorded.

### *Anopheles stephensi* species identification

All mosquitoes that were identified as recently blood fed were extracted (*n* = 5), and the rest of the mosquitoes were selected at random (*n* = 155). For the blood fed mosquitoes, DNA was extracted separately for the head/thorax and abdomens. Head and thoraces (*n* = 51), abdomens (*n* = 51), and whole mosquitoes (*n* = 58) were used to extract DNA using the DNeasy Blood and Tissue Kit or the DNA Micro Kit (Qiagen, Valencia, CA). Polymerase chain reactions (PCR) were conducted for each mosquito, targeting the *An. stephensi* specific nuclear internal transcribed spacer 2 (ITS2) locus and the universal mitochondrial cytochrome *c* oxidase subunit 1 (*COI*) [2, 23]. The final reagent components and concentrations for PCR were 1× Promega G2 HotStart Master Mix (Promega, Madison, WI), 0.5 mM for both primers, and 1 uL of DNA template. The endpoint assay targeted the ITS2 locus of *An. stephensi* [2]. The PCR temperature protocol was 95 °C for 1 min, 30 cycles of 95 °C for 30 s, 48 °C for 30 s, and 72 °C for 1 min; followed by 72 °C for 10 min. The PCR product was visualized via gel electrophoresis and a 522-base pair (bp) band was identified. Only *An. stephensi* samples would contain a band, so any samples that did not produce a band were not included in the sample set. The PCR temperature protocol consisted of 95 °C for 1 min, 30 cycles of 95 °C for 30 s, 48 °C for 30 s, and 72 °C for 1 min; followed by 72 °C for 10 min. *COI* PCR products were sequenced using Sanger technology by a commercial laboratory (Eurofins Genomics LLC, Psomagen).

### Analysis of *Plasmodium vivax Pv47* database sequences for geographically informative SNPs

An analysis of all available *Pv47* sequences was performed using CodonCode aligner v9.01 software to identify any SNPs or amino acid substitutions. Sequences of *Pv47* were first downloaded from the National Center for Biotechnology and Information (NCBI) GenBank, and previously generated *Pv47* sequences from Ethiopia were included (Additional file 1). The sequences were combined into a single FASTA file (opened in CodonCode) and the sequences were assembled into a contig. Sequences were organized by continent and differences in the sequences were visualized. Sequences were then translated into amino acids and the reading frame was selected on the basis of the reference amino acid sequence in NCBI (XM_001614197.1). Differences in amino acids were then visualized.

### *Plasmodium falciparum Pfs47 *and *Plasmodium vivax Pv47* primer design

The *Pfs47* gene from the reference *P. falciparum* genome (NCBI: taxid36329, strain Pf3D7) was used for the primers. The reference gene of *Pfs47* from the PF3D7 African strain of *P. falciparum* was put into Primer3Plus, with a target product indicating the SNPs previously identified as differentiating the continental origin of *P. falciparum* at bp positions 707 and 725. Primers were tested using a positive control of *P. falciparum* DNA. Positive control amplicons of *Pfs47* were sequenced to confirm the target region at 559 bp (Additional file 2: Table 2).

For the *Pfs47* nested primers, the amplified sequence of the un-nested primers was determined using NCBI Primer Blast. This product was uploaded to Primer3Plus and the area previously identified with SNPs was selected as a target. Potential primers were tested against the nonredundant and RefSeq mRNA databases in NCBI Primer Blast, and primers that did not also target *An. stephensi* DNA were selected. These primers were tested against the positive control DNA and sequenced to confirm the correct target. This produced a product of 531 bp (Additional file 2: Table 2).

A similar protocol was used to design the *Pv47* and *Pv47* nested primers as described above. The *Pv47* gene from the reference *P. vivax* genome (NCBI: taxid126793, XM_001614197.1) was used. The gene was put into NCBI Primer Blast, excluding *An. stephensi* DNA, with a target indicating where SNPs were identified to be in the described alignment. Potential primers were tested against the nonredundant and Ref Seq mRNA databases in NCBI Primer Blast and primers that did not also target *An. stephensi* DNA were selected. Primers were tested using a positive control of *P. vivax* DNA. Positive control amplicons were sequenced to confirm the target region at 581 bp (Additional file 2: Table 2).

The *Pv47* nested primers were created by first determining the amplified sequence of the un-nested primers using NCBI Primer Blast. The product was uploaded to Primer3Plus and the area previously identified to contain the SNPs was selected as a target. Potential primers were tested against the nonredundant and Ref Seq mRNA databases in NCBI Primer Blast, and primers that did not also target *An. stephensi* DNA were selected. These primers were tested against the positive control and sequenced for target region confirmation (a product of 414 bp) (Additional file 2: Table 2).

### *Plasmodium falciparum Pfs47* targeted sequencing

*Plasmodium falciparum Pfs47* was genotyped to detect parasite presence in *An. stephensi* samples. A positive control of positive *P. falciparum* human blood DNA extractions (provided by Dr. Eugenia Lo at Drexel University) served to verify successful amplification, alongside a negative control lacking genomic DNA to ensure no contamination was present. Un-nested PCR reactions were conducted initially to detect *P. falciparum Pfs47* presence before running nested PCR reactions. The *Pfs47* primers amplified a 559 bp un-nested fragment in *P. falciparum* (Additional file 2: Table 2). Un-nested protocol reagents and concentrations consisted of 1× Promega G2 HotStart Master Mix (Promega, Madison, Wisconsin, USA), 0.4 mM of primer, plus 4 uL of isolated DNA template. The cycling protocol was as follows: 95 °C for 1 min, 34 cycles of 95 °C for 1 min, 57 °C for 1 min, 72 °C for 1.5 min, and an extension step of 72 °C for 10 min. The nested protocol called for a second set of primers to selectively amplify a 531 bp fragment of *P. falciparum Pfs47* (Additional file 2: Table 2). The nested reaction was performed with 1× Promega G2 HotStart Master Mix (Promega, Madison, Wisconsin, USA) 0.4 mM of primer, plus 2 uL of the PCR product from the initial un-nested reaction. The cycling protocol was as follows: 95 °C for 10 min, 34 cycles of 95 °C for 1 min, 58 °C for 1 min, 72 °C for 1 min, followed by 72 °C for 5 min.

### *Plasmodium vivax Pv47* targeted sequencing

PCR was conducted to amplify the *Pv47* gene for the detection of *P. vivax* in mosquito samples. A positive control of positive *P. vivax* human blood DNA extractions (provided by Eugenia Lo at the University of North Carolina at Charlotte) was included, alongside a negative control lacking genomic DNA to ensure no contamination was present. An un-nested protocol was used to detect the presence of *P. vivax Pv47*, followed by a nested protocol.

*Pv47* primers amplified a 581 bp un-nested fragment in *Plasmodium vivax* (Additional file 2: Table 2). The un-nested protocol reagents and concentrations consisted of 1× Promega G2 HotStart Master Mix (Promega, Madison, Wisconsin, USA), 0.4 mM of primer, plus 4 uL of isolated DNA template. The cycling protocol was as follows: 95 °C for 1 min, 34 cycles of 95 °C for 1 min, 58 °C for 1 min, 72 °C for 1.5 min, and an extension step of 72 °C for 10 min. The nested protocol called for a second set of primers to selectively amplify a 414 bp fragment of *P. vivax Pv47* (Additional file 2: Table 2). The nested reaction was performed with 1× Promega G2 HotStart Master Mix (Promega, Madison, Wisconsin, USA), 0.4 mM of primer, plus 2 uL of the PCR product from the initial un-nested reaction. The cycling protocol was as follows: 95 °C for 10 min, 34 cycles of 95 °C for 1 min, 60 °C for 1 min, 72 °C for 1 min, followed by 72 °C for 5 min. All PCR products were run on a 2% agarose gel and visualized. Positive samples were sequenced via Sanger Sequencing at a commercial laboratory (Eurofins Genomics LLC, Psomagen).

### *Anopheles stephensi P47Rec* targeted sequencing

PCR was conducted to amplify the P47 receptor in *An. stephensi* samples. Overall, 20 mosquitoes were randomly selected from Kebridehar, Ethiopia (2018), 20 from Semera, Ethiopia (2018), 20 from Dire Dawa, Ethiopia (2018), 13 from Lawyacado, Somalia (2021), and 20 more from Dire Dawa (2022). *P47Rec* primers amplified a 688 bp fragment including the second and third exon of the protein (F: 5′-TGGCAAATGACTAACGTGGA-3′, R: 5′-GTGTTGCCAGTTCGCTGTAA-3′). The cycling protocol was as follows: 95 °C for 1 min, 34 cycles of 95 °C for 1 min, 58 °C for 1 min, 72 °C for 1.5 min, and an extension step of 72 °C for 10 min. All PCR products were run on a 2% agarose gel and visualized. All samples were sequenced via Sanger Sequencing at a commercial laboratory (Psomagen).

### *Pfs47 *and *Pv47* sequence analysis

The haplotypes for the sequences that indicated multiple nucleotides read at a single position were phased out using DNAsp v 5.10.01. The FASTA file containing cleaned sequences for both genes were uploaded into DNAsp and phased using Markov chain Monte Carlo (MCMC) standard options (100 iterations, 1 thinning interval, 100 burn-in iterations). A new FASTA file was exported and each sample that had multiple haplotypes was denoted as haplotype 1 or 2 after the sample name. Sequences were also translated using CodonCode v9.0.1 and the reading frame was selected to match the protein coding of Pfs47 and Pv47 on NCBI (PV077639-PV077662).

### *COI* phylogenetic analysis

Phylogenetic analysis was first performed on the *COI* sequences of *Pfs47* or *Pv47* positive samples with an outgroup of *An. maculatus* (KT382822). Phylogenetic analyses were estimated using a maximum likelihood approach with RaxML [24]. The GTR GAMMA option that uses the general time-reversible model of nucleotide substitution with the gamma model rate of heterogeneity was used. In total, 1000 runs were completed with rapid bootstrap analysis. The RAxML output was viewed in FigTree with a root at the outgroup [25].

### Isolation of *Pfs47*, *Pv47*, and *P47Rec* sequences from whole genome data

The extraction of *Pfs47*, *Pv47*, and *P47Rec* sequences followed a comprehensive bioinformatics pipeline. First, the raw FASTQ files were obtained from the databases MalariaGen and NCBI [26, 27]. These sequences were aligned to their respective reference genomes (PF3D7 for *Pfs47*, XM_001614197 for *Pv47*, and UCI_ANSTEP_V1.0 for *P47Rec*) using Bowtie2, ensuring precise mapping of reads. The aligned sequences were converted into BAM files using Samtools to create binary alignments. Subsequently, Bcftools was used for variant calling, employing “mpileup” to aggregate aligned reads and “call” to identify potential variants. Variant filtration ensured high-quality data, with filters applied for Phred-scaled quality scores greater than 30, read depths above 10, and variant frequencies above 1%.

The variants were processed to generate FASTA sequences after quality filtering. These sequences were further aligned using MAFFT to prepare them for downstream analyses, including evolutionary or functional assessments [28]. This methodical process ensured accurate extraction and alignment of the target gene sequences from complex whole genome datasets.

### Minimum spanning network analysis

Sequences from online databases such as NCBI GenBank and MalariaGen were processed as described and aligned with all *Pfs47* or *Pv47* sequences in this study using CodonCode Aligner v9.0.1 and exported into a FASTA file. This FASTA file was converted to rphylips and the data was copied into Microsoft Excel. Random haplotype names were given to every unique haplotype for both *Pfs47* and *Pv47*. The frequencies and sequences of each haplotype were formatted into a .nex file to be imported in popart [29]. A minimum spanning network was then created using standard settings and an epsilon value of 0. Haplotypes were colored according to the continent and the presence of *An. stephensi*.

## Results

### *Plasmodium *detection in wild *Anopheles stephensi* from an outbreak

*P. falciparum* and *P. vivax* DNA were detected in 10.625% (17/160) of samples screened. More samples were positive for *Pfs47* than *Pv47* (10/17 and 7/17, respectively), and more whole-body extractions were positive than head/thorax or abdomen extractions (10/17 and 7/17, respectively).

### *Pfs47* diversity and similarity with African and Asian sequences

Overall, 10 out of 160 samples tested were positive for *Pfs47* (6.25% positive, 10/160): 2 from abdomen extractions, 2 from head and thorax extractions, and 6 from whole body extractions. Analysis of the 531 bp sequence revealed nine segregating sites resulting in eight haplotypes, with one haplotype predominately present (Hap 1: 53.33%, Hap 2: 6.67%, Hap 3: 13.33%, Hap 4: 6.67%, Hap 7: 6.67%). Some chromatograms showed two nucleotides read in the same position indicating either heterozygosity at that position or infections with multiple genetically distinct *P. falciparum* strains. Two whole body extractions were positive for both *Pfs47* and *Pv47*.

We genotyped geographically informative SNPs at positions 707 and 725 to determine the continental origin of the *P. falciparum* detected in *An. stephensi*. All sequences were identified to have a C–C haplotype at SNPs 707 (236T) and 725 (242S), which is indicative of the African haplotype (Additional file 2: Table 3). After phasing out heterozygous samples, there were 14 individual *Pfs47* sequences. In total, nine different amino acid changes were detected from the PF3D7_1346800 reference: F128L (*n* = 1, nucleotide 382), L172S (*n* = 1, nucleotide 515), P194H (*n* = 14, nucleotide 581), S212R (*n* = 1, nucleotide 636), D213G (*n* = 2, nucleotide 638), G228A (*n* = 1, nucleotide 683), G228V (*n* = 1, nucleotide 683), K220E (*n* = 1, nucleotide 658), and L248I (*n* = 2, nucleotide 742). Specific combinations of these amino acid changes can be seen in Table 1. The sample containing the S212R mutation had no other amino acid substitutions from the reference. The most common genotype contained the P194H mutation and the I248L mutation. All the Ethiopian samples, including the positive control, had an amino acid substitution from the reference sequence (PF3D7_1346800) at the 194th position, changing from P to an H. Overall, 42 segregating sites were present in the whole protein (Additional file 2: Table 4). No amino acids substitutions were observed involving cysteine residues.

**Table 1:** Amino acid substitutions in *Pfs47* sequences from Dire Dawa in reference to the Pf3D7 reference genome (gene ID: 814213)

Sample ID	Amino acid substitutions								Haplotype
S220123_1	–	–	P194H	S212R	–	–	–		1
S220123_2	–	–	P194H	–	–	–	–		2
S220282_1	–	–	P194H	–	D213G	G228A	–		3
S220282_2	–	–	P194H	–	D213G	G228V	–		3
S220267_2	F128L	L172S	P194H	–	–	–	–	I248L	4
S220247	–	–	P194H	–	D213G	–	–		5
S220246_2	–	–	P194H	–	–	–	K220E	I248L	6
S220246_1	–	–	P194H	–	–	–	–	I248L	7
S220223	–	–	P194H	–	–	–	–	I248L	7
S220267_1	–	–	P194H	–	–	–	–	I248L	7
S220230	–	–	P194H	–	–	–	–	I248L	7
S220108	–	–	P194H	–	–	–	–	I248L	7
S220098	–	–	P194H	–	–	–	–	I248L	7
S220078	–	–	P194H	–	–	–	–	I248L	7

There are eight amino acid haplotypes, as well as nucleotide haplotypes present in Dire Dawa. The number one or two after a sample ID indicates the presence of heterozygosity at that nucleotide position, resulting in two genotypes after phasing. Amino acid substitution P194H was present in all samples, I248L in over half (*n* = 9), and D214G in three samples. F128L, L172S, S212R, G228A, G228V, and K220E all occurred once. “–” identifies the same sequence as the reference

**Fig. 1:**
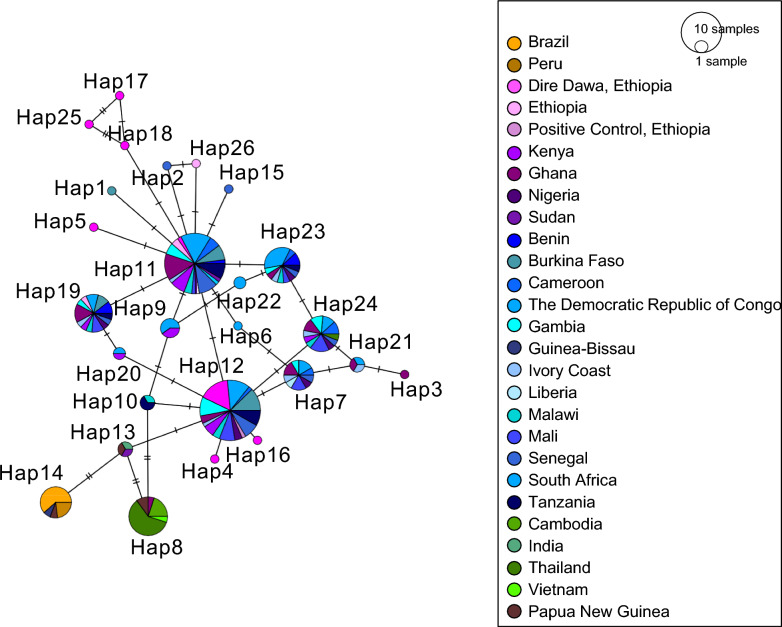
Minimum spanning network of *Pfs47* sequences from NCBI Genbank, MalariaGen database, and Dire Dawa. The number of sequences is represented by the size of circle, and single point mutations between haplotypes are represented by hash marks. Samples produced in this study are colored in bright pink. Countries where invasive *An. stephensi* has been detected are shown in purple, all other countries in Africa are shown in blue, countries in Asia are shown in green, countries in South America are shown in orange, and Papua New Guinea is shown in brown. Dire Dawa shares a major haplotype with other countries in Africa (Hap11 and Hap12) and has some unique haplotypes as well (Hap25, Hap17, Hap18, and Hap5). Hap13 is connected to a major African haplotype (Hap12) and the South American and Asian haplotypes (Hap14 and Hap8, respectively), and notably contains sequences from Sudan, India, and Papua New Guinea. Ghana shares a haplotype with the major Asian haplotype (Hap8). Thailand shares a haplotype with African sequences (Hap24)

Minimum spanning network (MSN) analysis of the 312 bp sequence shows an extensive network of diversity of the domain 2 region of the *Pfs47* in Africa (Fig. 1). Here, haplotypes are defined as nucleotide sequences that have at least one difference, where the presence of multiple nucleotide differences between haplotypes indicates more divergence. We found that Hap11 and Hap12 had the highest frequency. While there were continentally differentiated haplotypes, *Pfs47* haplotypes were shared across countries in Africa (e.g., Hap11 and Hap12). Dire Dawa exhibits six individual haplotypes not observed elsewhere (Hap4, Hap16, Hap17, Hap18, and Hap25). Ghana shares a haplotype with Asian sequences (Hap8), and Guinea Bissau shares a haplotype with South American sequences (Hap14). Hap13 bridges the South American and Asian haplotypes (Hap14 and Hap8), as well as a major African haplotype (Hap12). Hap 13 also encompasses *P. falciparum* from India, Sudan, and Papua New Guinea. The major Asian haplotype (Hap8) has two nucleotide differences compared with a haplotype present in Tanzanian and Malawian *P. falciparum* strains (Hap10), as well as in East Africa. South American *Pfs47* (Hap14) is conserved and distinct from other continents, and sequences from Oceania share haplotypes with all three continents (Hap8, Hap13, and Hap14) (Fig. 1).

### *Pv47* alignment

The alignment of available *Pv47* sequences from NCBI GenBank revealed three SNPs that differentiated South America from Asia and Africa at positions 1221, 1222, and 1230 of sequence JQ435617, and from India 100% of the time (Additional file 2). There were no SNPs identified in the portion of *Pv47* available that differentiated all continents 100% of the time.

### *Pv47* diversity and similarity with African and Asian sequences

Seven samples were positive for *Pv47*: three from abdomen extractions and four from whole body extractions (4.37% positive, 7/160). Six haplotypes were present, with one predominant haplotype present in four of seven samples (Hap 1: 44.44%, Hap 2, 3, 4, 5, and 6: 11.11%). Two *Pv47* positive samples indicated either heterozygosity or multiple different *P. vivax* strains.

**Fig. 2:**
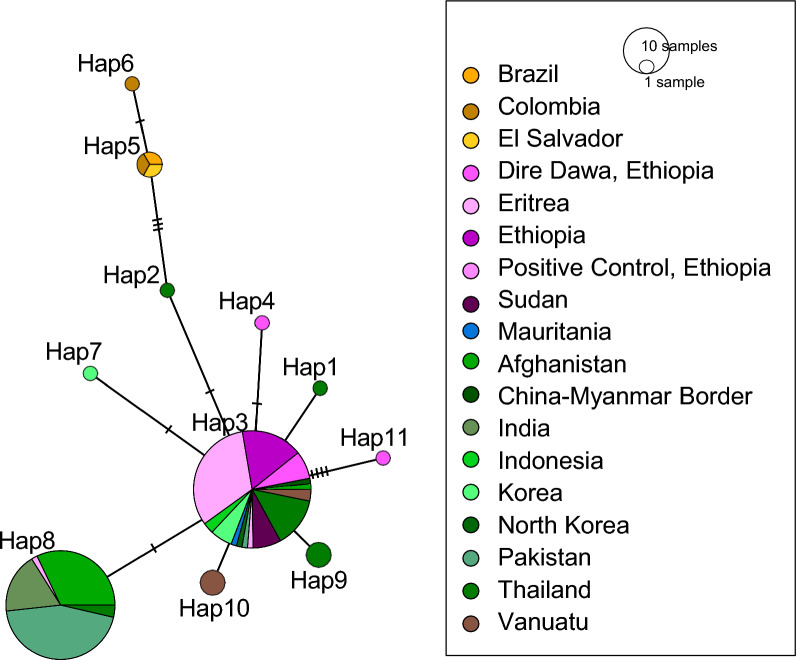
Minimum spanning network of *Pv47* sequences from NCBI and Dire Dawa. The number of sequences is represented by the size of circle, and single point mutations between haplotypes are represented by hash marks. Samples produced in this study are shown in bright pink. Countries where invasive *An. stephensi* has been detected are shown in purple, all other countries in Africa are shown in blue, countries in Asia are shown in green, countries in South America are shown in orange, and Vanuatu is shown in brown. Hap3 represents most sequences from Africa and Asia, although Hap8 is also a major Asian haplotype that is also shared with the positive control from Ethiopia. South American sequences are present in Hap6 and Hap7. Samples from Dire Dawa share similarity with Hap3 but have two unique haplotypes (Hap11 and Hap4)

There is a single haplotype that encompasses most of the samples from Africa and Asia (Hap3). Dire Dawa has another haplotype that is differentiated from other Africa haplotypes (Hap11). South American haplotypes (Hap5 and Hap6) are similar to haplotypes in Thailand (Hap2) but with several nucleotide differences separating them. Sequences from Oceania share the major African and Asian haplotype (Hap3) and have one unique haplotype (Hap10) (Fig. 2). A second major Asian haplotype (Hap8), mostly indicative of India, Pakistan, and Afghanistan, shares sequence similarity with one sequence from Ethiopia (Fig. 2). All the Pv47 samples had amino acids changes from the reference sequence (XM001614197.1): F22L, F24L, and K27E. These amino acid substitutions have been observed previously in Asian sequences, although there are 21 segregating sites in the whole protein globally (Additional file 2: Table 5) [21]. No amino acid substitutions were observed involving cysteine residues.

### *P47Rec* diversity

**Fig. 3:**
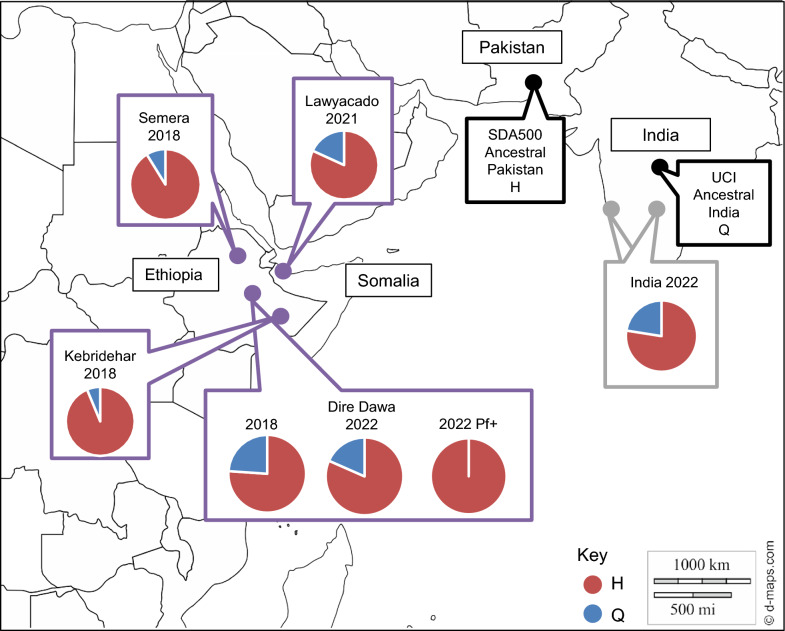
Allele frequency of the amino acids histidine or glutamine at position 53 in different samples and reference genomes of *An. stephensi*. Invasive populations of *An. stephensi* are outlined in purple, native in gray, and reference in black. Allele frequency of H is denoted by red in the pie charts, and the frequency of Q is denoted by blue

We sequenced the entire *P47Rec* gene in a subset of samples to evaluate *An. stephensi* diversity in multiple populations across time that have demonstrated increased malaria transmission or significance in gene flow within the Horn of Africa [16, 29]. The P47Rec amino acid sequences were the same in all *An. stephensi* from Ethiopia except for one amino acid in the second exon (amino acid position 53), and the second exon was sequenced for the rest of the samples. At this position, samples were either homozygous for histidine (53H) or glutamine (53Q), or were heterozygous, consistent with *An. stephensi* reference sequences. In 2018, Kebridehar had two heterozygous samples (2/16) and the rest were homozygous for H (14/16), Semera had three heterozygous samples (3/17) and the rest were homozygous for H (14/17), and Dire Dawa had two samples homozygous for Q (2/23), seven heterozygous samples (7/23), and the rest homozygous for H (14/23). In Lawyacado, one sample was homozygous for Q, two were heterozygous (2/11), and the rest were homozygous for H (8/11). In Dire Dawa in 2022, all *P. falciparum* positive samples from this study were homozygous for H (6/6), with samples positive for both *P. falciparum* and *P. vivax* being both homozygous for H (1/2) and heterozygous (1/2). The nonpositive samples from Dire Dawa 2022 had one sample homozygous for Q (1/19), five heterozygous samples (5/19), and the rest were homozygous for H (13/19). Samples from India had homozygosity for both H and Q (12/20 HH, 1/20 QQ), and seven heterozygous samples (7/20) (Fig. 3; Additional file 2: Table 6).

### *Pfs47*/*Pv47* and *Anopheles stephensi COI* diversity

*COI* haplotypes were evaluated in the mosquitoes to investigate any *An. stephensi* lineages specifically infected by *P. falciparum* or *P. vivax*. Four previously identified *COI* haplotypes from Ethiopia were present in the sample set. Overall, 74 samples were identified as haplotype 2, 10 as haplotype 4, 1 as haplotype 1, and 23 as haplotype 3. Haplotype 2 samples had the most positive samples but were also the most prevalent. Haplotypes 2, 3, and 4 were observed in malaria positive samples, with the most positives being observed in haplotype 2. Haplotype 2 was the most predominant haplotype, so a larger sample size is needed to determine any correlation (Additional file 2: Fig. 1).

## Discussion

The data presented here provides novel evidence of both *P. falciparum* and *P. vivax* in invasive *An. stephensi* during the 2022 malaria outbreak in Dire Dawa, Ethiopia, with *P. falciparum* of African origin. The relatively high detection rate of 6.21% for *P. falciparum* and 4.37% for *P. vivax* in these mosquitoes is consistent with an outbreak scenario [30]. More whole bodies were positive than head and thorax or abdomens, likely because more were screened. In *Pfs47*, the SNPs at locations 707 and 725 of the gene revealed that the *P. falciparum* in these *An. stephensi* populations exhibit the genotype that is conserved in 99.8% of African *Pfs47* [12]. All *Pfs47* haplotypes identified in this study were either connected with or shared haplotypes with other African sequences, further confirming the African origin of the *P. falciparum* in these *An. stephensi*. Additional support relates to previously identified markers for artemisinin and diagnostic resistance in *P. falciparum* during the outbreak, where two mutations were identified that have previously been found in the Horn of Africa [8]. Together, these data provide evidence to support that the *P. falciparum* transmitted by *An. stephensi* during the outbreak was of African origin, rather than imported South Asian *P. falciparum*, and emphasizes a variation among anophelines and their specificity.

While most of the African *P. falciparum* haplotypes clustered separately from other continental groups, the MSN analysis for *Pfs47* revealed a connection between Asian and African haplotypes. The predominant Asian *Pfs47* haplotype (Hap8) has the closest connection to Hap 10, which includes Tanzania and Malawi, and Hap13, which includes Sudan and India. Population genetics analysis of Sudanese *An. stephensi* have shown considerably high genetic variation consistent with being one of the older invasive *An. stephensi* populations [30]. Finding shared *Pfs47* haplotypes with India highlights the connection between Sudan and South Asia, and the role the location may have played in the earlier introduction of *An. stephensi*. Further studies on *P. falciparum* diversity across the *An. stephensi* range could provide additional insight into the potential for importation.

In other vectors, such as local *An. gambiae* sensu stricto (s.s), *P. falciparum* infection is based on a lock-and-key theory where the parasite has a variable region in *Pfs47* that allows for evasion of the mosquitoes’ immune system [11, 13]. In these wild *An. stephensi*, eight *Pfs47* haplotypes were observed, with some samples containing the I248L mutation previously identified. A study found that in *An. gambiae *sensu stricto, this mutation caused 100% melanization of parasites, but in the *An. stephensi* SDA500 there were 100% live parasites, indicating that singular amino acid changes, whether conservative or not, do not impact infection. Presumably, with both the isoleucine and leucine present in this *An. stephensi* parasite population, it seems as though singular amino acid mutations may determine infection in local African vectors but do not define infection in invasive *An. stephensi*. Nucleotide diversity in the *Pfs47* gene across human infections has been observed previously within a single country of origin. The presence of multiple amino acid haplotypes within a single vector in one city suggests a more ambiguous interaction between P47 and P47Rec than in *An. gambiae* complex mosquitoes [12, 17]. Significantly, no amino substitutions were observed involving cysteine residues in either Pfs47 or Pv47, indicating the continued possibility for these genes to be an effective TBV target in this region.

The P47Rec of other anopheline vectors with well-studied Pfs47–P47Rec relationships, such as *An. gambiae*, are 100% conserved across the African continent [19]. This supports the lock-and-key theory, where *An. gambiae*–*P. falciparum* interaction is very specific. We see in these invasive *An. stephensi* that there is genetic diversity across time and across the Horn of Africa, supporting the idea that *An. stephensi* has a more ambiguous *Pfs47* interaction with *P. falciparum* [9]. Moreover, all genotypes present in the *P. falciparum*-positive *An. stephensi* were homozygous for histidine. When both *P. falciparum* and *P. vivax* were present, glutamine was only present in heterozygous form, potentially indicating that population level diversity in *An. stephensi* may influence variation within *Pfs47*. A larger sample size accompanied by experimental infection studies is needed to make definitive conclusions.

There is limited data available on *P. vivax Pv47* related to basic biology, compatibility with different vectors, and geographical specificity. Using data from NCBI GenBank, we were unable to identify SNPs or amino acid changes that differentiate continental origin between Africa and Asia, agreeing with a previous study on global *P. vivax* found in both African and Asian samples [15]. Dire Dawa *Pv47* exhibits more diversity than other African sequences, although there is only representation from three African countries. There is a distinct haplotype present in Dire Dawa (Hap4) that shows distant relatedness to the rest of African and Asian Hap3. The amount of *P. vivax* positive mosquitoes was almost equal to those positive with *P. falciparum*, even two mosquitoes possibly coinfected with both, potentially indicating equal levels of transmission of both forms of human malaria during the outbreak. Previously published data on the outbreak identified most of the human samples were positive for *P. falciparum*, so there is likely some variable impacting the discordance in detection levels: either temporally related or behavior related [8].

Overall, the story of *Pfs47* mediated *Plasmodium* infection in *An. stephensi* seems to be much more complex than in native African anophelines. To this end, we present compelling data on both the vector and the parasite, *P47* and *P47Rec*, in which there is more diversity present in a singular species than observed in local malaria vectors such as the *An. gambiae* complex. The results of this study emphasize that invasive *An. stephensi* may not strictly follow the lock-and-key model where a high number of conservative and nonconservative Pfs47 amino acid changes are observed, as well as a receptor that does not appear to be conserved, as previously noted [9]. This is indicated by the agreement with laboratory data of *An. stephensi* (SDA500), where there are multiple amino acid changes in domain 2 of Pfs47 that did not affect *P. falciparum* infectivity. While we cannot confirm that the *Plasmodium* DNA detected here was not of melanized parasites, the discovery of these diverse *P. falciparum Pfs47* sequences are still alarming, considering it could be suggestive that wild *An. stephensi* is like the laboratory strain in the P47–P47Rec system. Limited information can be concluded about *Pv47.*; however, it appears *Pv47* from Africa and Asia are not clearly delineated, which could be explained by a shared recent ancestry [15]. If P47 is a marker of compatibility in *P. vivax*, as it is in *P. falciparum*, then the similarity in the Asian and African ligand would indicate the ease of *P. vivax* infection by both invasive and local anophelines. This information provides evidence for the need for further study in *An. stephensi* to determine what other genes are involved in determining both *P. falciparum* and *P. vivax* infection in these invasive populations. These genes could potentially become diagnostic markers for permissiveness of invasive *An. stephensi* to *Plasmodium* or the propensity for *An. stephensi* to cause an outbreak.

## Conclusion

We investigated the continental origin and overall diversity of* Plasmodium* in invasive *An. stephensi* collected during a malaria outbreak. We were able to identify high levels of both *P. falciparum* and *P. vivax* in these *An. stephensi*, with the *P. falciparum* being of African origin. Diverse haplotypes of P47 were observed in both *P. falciparum Pfs47* and *P. vivax* *Pv47.*, with unique haplotypes present that have not previously been observed. Targeted sequencing of the corresponding P47Rec revealed a single amino acid change, not typical in anophelines. Overall, this study revealed a complex interaction between* Plasmodium* and invasive* An. stephensi*. These findings highlight the need for further investigation into the applicability of these genes for the diagnosis of permissiveness of *An. stephensi* to* Plasmodium* or the propensity of *An. stephensi* to cause an outbreak.

## Supplementary Information

Additional file 1.

Additional file 2.

## Data Availability

All data generated or analyzed during this study are included in this published article and its supplementary information files. Sequences for *Pfs47* and *Pvs47* are published in NCBI GenBank under accession numbers PV077639-PV077662. *P47Rec* sequences are under accession numbers PV100900 – PV101033 and PV179422-PV179438.

## Abbreviations

SNPs: Single nucleotide polymorphisms
aa: Amino acid
P47Rec: P47 receptor
TBV: Transmission blocking vaccine
MSN: Minimum spanning network
bp: Base pair
PCR: Polymerase chain reaction
ITS2: Internal transcribed spacer 2
*COI*: Cytochrome *c* oxidase subunit 1
NCBI: National Center for Biotechnology Information
MCMC: Markov chain Monte Carlo
